# Urban Ecosystem Services for Resilience Planning and Management in New York City

**DOI:** 10.1007/s13280-014-0509-8

**Published:** 2014-04-17

**Authors:** Timon McPhearson, Zoé A. Hamstead, Peleg Kremer

**Affiliations:** 1 Tishman Environment and Design Center, The New School, 79 Fifth Avenue, 16th Fl., New York, NY 10003 USA; 2 Milano School of International Affairs, Management and Urban Policy, The New School, 72 Fifth Avenue, New York, NY 10003 USA

**Keywords:** Urban ecosystem services, Urban planning, Management, Scale, New York City

## Abstract

We review the current state of knowledge about urban ecosystem services in New York City (NYC) and how these services are regulated, planned for, and managed. Focusing on ecosystem services that have presented challenges in NYC—including stormwater quality enhancement and flood control, drinking water quality, food provisioning and recreation—we find that mismatches between the scale of production and scale of management occur where service provision is insufficient. Adequate production of locally produced services and services which are more accessible when produced locally is challenging in the context of dense urban development that is characteristic of NYC. Management approaches are needed to address scale mismatches in the production and consumption of ecosystem services. By coordinating along multiple scales of management and promoting best management practices, urban leaders have an opportunity to ensure that nature and ecosystem processes are protected in cities to support the delivery of fundamental urban ecosystem services.

## Introduction

Cities are complicated social–ecological systems with both tightly and loosely connected components interacting dynamically over space and time (Pickett et al. 2001) making resilient, equitable, sustainable cities difficult to achieve. Urban resilience depends on the urban system’s ability to simultaneously maintain social and ecological functions (Alberti et al. 2003). Ecosystem services provide an important framework for linking ecological infrastructure to social infrastructure in the city, with the potential to benefit humans and ecosystems. Designing, planning, and managing complex urban systems for human health and well-being require urban ecosystems to be resilient to systemic change, and to be managed sustainably to provide critical ecosystem services reliably over time.

Nature in cities plays a crucial role in urbanized systems as the ecological basis for human–nature interactions and the production of urban ecosystem services (Bolund and Hunhammar 1999; TEEB 2011; Gómez-Baggethun et al. 2013). Since the early days of urban planning, planners have sought various means of incorporating nature into the city and preserving the surrounding landscape (Jacobs 1961; Howard 1965; McHarg 1992). Many early landscape architects, notably Fredrick Law Olmsted, sought not only to improve the appearance of the city, but also to improve health and provide areas for rest and recreation for the crowded urban population (Hough 2004). In addition to the cultural benefits that ecosystem functions provide to urban residents, other services such as clean water and clean air are also crucial to health and well-being of urban populations. Here we review the current knowledge of urban ecosystem services in New York City (NYC) and their inclusion in current plans and policies as a foundation for the development of urban resilience planning, policy, and management in the city.

## The Social–Ecological System of NYC

The New York Metropolitan region is a classic example of a complex social–ecological system (Cadenasso et al. 2007). Situated along the northeast coast of the United States, the New York Metropolitan region, with unparalleled ethnic and social diversity, encompasses a dense urban core, surrounded by suburban and exurban housing development. New York became the world’s first global megacity in 1950 when its population reached 10 million (Chandler 1987) and still ranks as one of the world’s largest megacities with 22.2 million people living in the metropolitan region (U.S. Census Bureau 2010) and 8.3 million residents within the municipal city (NYC) that includes the boroughs of Manhattan, Queens, Bronx, Brooklyn, and Staten Island. NYC is the most populous and dense (10 630 residents km^−2^) of all U.S. municipalities (Mackun and Wilson 2010), and has a higher percentage of open space than any other major U.S. city (The Trust for Public Land 2011). NYC’s land area covers ~790 km^2^ with open space making up 27 % of the city. The rest of the city land area includes 27.3 % in low-density residential use, 12.2 % in multi-family residential use, 7.1 % transportation/utility, 6.9 % public facilities and institutions, 5.8 % vacant land, 4 % commercial/office, 3.6 % industrial/manufacturing, 3 % in mixed residential and commercial, 1.3 % parking facilities, and 1.8 % no data (New York City Department of City Planning 2013).

The population density of the city is matched by its cultural diversity. Thirty-six percent of the city’s population is foreign-born (Lobo and Salvo 2004) and NYC continues to be the leading gateway for immigrants to the U.S. (Monger and Yankay 2011). Over 800 languages are spoken in NYC, the most linguistically diverse city in the world (Roberts 2010).

**Fig. 1:**
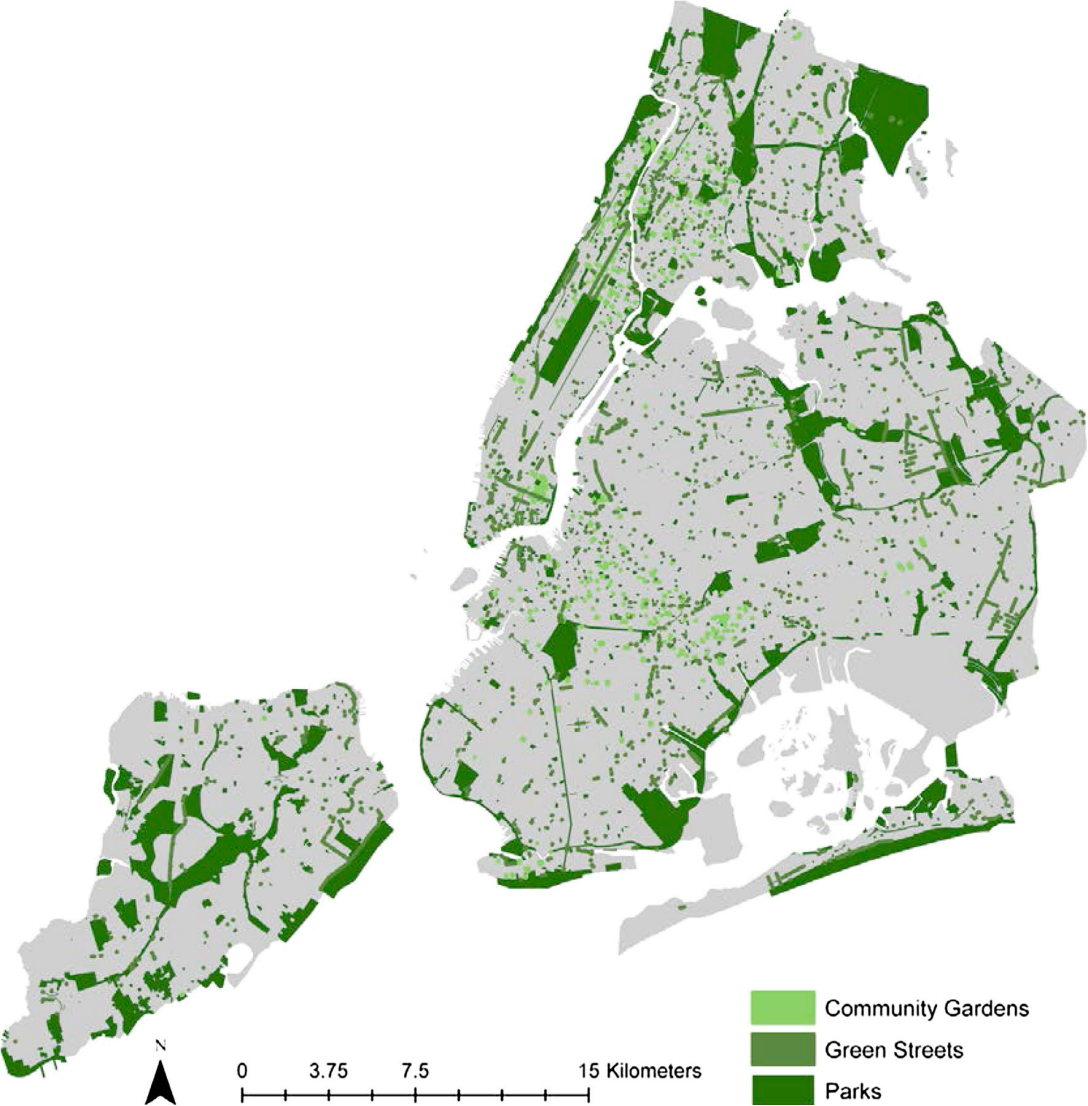
New York City Green Infrastructure. Green infrastructure includes city parks, green streets, and community gardens. Data Sources: NYC Department of Parks & Recreation and NYC Department of Information Technology & Telecommunications

Ecologically, NYC lies at the confluence of several waterways that form one of the world’s largest natural harbors used extensively for import and export activities (Kurlansky 2006). Thirty-five percent of the city’s area is water, and includes 23 km of public beaches. Throughout the five boroughs of NYC, there are 110 km^2^ of city parkland—nearly 40 % of which is still natural—harboring freshwater wetlands, salt marshes, rocky shorelines, beaches, meadows, and forests. The diverse ecosystems of NYC include 6.7 km^2^ of freshwater wetlands, 5.8 km^2^ of grassland communities, 20.8 km^2^ of forest, 6 km^2^ of salt marsh, approximately 2 million trees in landscaped parks, 630 000 street trees, and over 2500 green streets (Fig. 1). In total there are over five million trees in NYC (Nowak et al. 2007) with tree canopy covering 21 % of land area. Still, NYC is expected to add nearly one million new urban residents in the next 20 years (City of New York 2006), introducing new challenges for managing local ecosystems to meet increased demand for fundamental ecosystem services in the city, including stormwater absorption, climate regulation, air pollution removal, noise mitigation, food production, drinking water, and recreation.

## Materials and Methods

In this paper, we review and analyze the current knowledge of the state of ecosystem services in NYC, and the extent to which ecosystem services are managed, regulated, and planned. First, we identified provisioning, regulating, and cultural services that are consumed by residents in NYC. We then conducted a literature review including both the peer reviewed and practitioner literature on topics related to ecosystem services and management in NYC and the region, collecting the following information: (1) the scale of production (whether local, regional, or global) of each ecosystem service; (2) the production unit(s), or ecosystem type in which the ecosystem service is produced; and (3) the scale(s) of management, regulation, or planning (whether federal level, state level, regional level, city level, or by community groups and non-profits). For several ecosystem services that have recently been particularly important and challenging to provide within NYC, we closely examine the context and challenges surrounding each, and describe their specific management regimes. These services include stormwater quality and flood control, drinking water supply and quality, and food provisioning. We then analyze matches and mismatches between the scale of production and scale of management, regulation, or planning for NYC ecosystem services. For ecosystem services not produced at the city level, we highlight instances in which entities with jurisdiction over the scale of production manage ecosystem services in cooperation with entities that have jurisdiction over the scale at which ecosystem services are consumed (city level).

## Ecosystem Services in NYC

### Urban Ecosystem Services Depend on Biodiversity

Biodiversity of and within urban ecosystems is integral to ecosystem functioning and the provision of ecosystem services to urban residents (Gómez-Baggethun et al. 2013). NYC is rich in biodiversity, though quality and quantity of both aquatic and terrestrial habitat for biodiversity have decreased over the years as a result of development, land use change, population growth, changing priorities in urban planning and management, climate change, and invasive species. Since biodiversity provides the basic ecological structure and functioning from which ecosystem services are produced, regular biodiversity assessment as well as how ecosystem functioning changes over space and time is central to planning, policy, and management for urban ecosystem services. When urban green space is undermined by development or competing planning priorities, as has been the case historically in NYC, the importance of existing urban nature, its ecological functioning, connectivity, and ability to provide ecosystem services has to be carefully considered in the planning and design process (Yli-Pelkonen and Niemelä 2005). Though cities and urbanized regions depend on biodiversity and ecosystems to sustain human health and well-being (TEEB 2011), this relationship is not well understood for all ecosystem services, and the connection between biodiversity and human livelihoods has yet to become mainstream.

### Urban Ecosystems and Service Providing Areas

Ecosystem services refer to those ecosystem functions that are used, enjoyed, or consumed by humans, which can range from material goods (such as water, raw materials, and medicinal plants) to various non-market services (such as climate regulation, water purification, carbon sequestration, and flood control) (Crossman et al. 2013; Gómez-Baggethun et al. 2013). Ecosystem services have been categorized as supporting (e.g., biodiversity), provisioning, regulating, or cultural (TEEB 2011). In the past two decades in NYC, a variety of ecosystem services have been assessed in multiple planning, policy, and research contexts. Early ecosystem service assessment efforts include the economic valuation of watershed quality and water provision (New York City Watershed Memorandum of Agreement 1997; Pires 2004; National Research Council 2000; NYC Environmental Protection 2010a; Watershed Agricultural Council 2011; New York City Department of Environmental Protection 2012) and economic valuation of forest ecosystem services (U.S. Environmental Protection Agency 2001; Grove et al. 2006; Nowak et al. 2007). More recent efforts include planning and legislation aimed at expanding and enhancing ecosystem services to improve the health and well-being of NYC residents. The most prominent example is the recent 20-year economic and environmental sustainability plan, PlaNYC, which includes 132 initiatives (McPhearson et al. 2013). Below, we review several ecosystem services of particular importance in NYC, including the regulating service of stormwater absorption, provisioning of food and drinking water, and the cultural service of recreation. We focus on these ecosystem services because they represent each category of ecosystem service (excluding supporting services provided by biodiversity) and because recent policy, planning, and management efforts in NYC have targeted these services.

Ecosystem services consumed by New Yorkers are produced at multiple spatial scales—from local to global—and are managed at the federal, state, regional, and local levels by an array of governmental agencies, community groups, and non-profits. A majority of ecosystem services surveyed in this review are produced at the local or regional level. Locally produced ecosystem services include food production in urban gardens, runoff mitigation in urban forests and other green infrastructure, and local climate regulation by urban forests and street trees. Regional ecosystems produced beyond the city’s municipal boundaries provide critical ecosystem services to city residents, including drinking water supply and drinking water quality regulation, air purification, food production, recreation, and more. Some ecosystem services, such as the supply of food, are generated at all spatial scales from local to global. Table 1 presents a summary of the literature review of ecosystem services in NYC and the region. Service providing units (SPUs) (Kremen 2005) denote the type of ecosystem and environmental conditions that support the production of ecosystem services. These include agricultural fields, wetlands and other blue infrastructure, regional forests and other kinds of urban green infrastructure including parkland, cemeteries, street trees, vegetated vacant land, and other open space. Depending on their scale of production, ecosystem services are produced by different SPUs. For example, while it is likely that a majority of food provision services are provided by agricultural land across the US and globally, the supply of water is largely provided by one regional watershed.

**Table 1:** Ecosystem Services in New York City. The table presents a summary of the literature review of major studies, policies, and plans of ecosystem services in NYC and is organized by the type of ecosystem service (provisioning, regulating, and cultural), the scale at which each ecosystem service is produced, the relevant service providing units, and the scale at which each ecosystem service is managed, regulated, or strategically planned for in NYC

Ecosystem service	Scale of production			Production unit (ecosystem type, species)	Scale of management, regulation and planning				
	Local	Regional	Global		Federal	State	Regional	City	Community groups and other non-profits
Provisioning									
Food: produce and crops	✓^(1)^	✓^(2)^	✓^(3)^	Local: private gardens, community Regional\global: agriculture fields, gardens			✓^(17)^	✓^(18)^	✓^(19)^
Food: livestock		✓^(4)^		Agriculture fields					
Food: seafood		✓^(5)^	✓^(5)^	Lakes, rivers, wetlands, estuaries, oceans					
Drinking water supply		✓^(6)^		Watershed	✓^(20)^	✓^(21)^	✓^(22)^	✓^(23)^	
Wood and fiber		✓^(7)^		Forest		✓^(24)^			
Regulating									
Drinking water quality enhancement		✓^(8)^		Watershed forest	✓^(25)^	✓^(26)^	✓^(27)^	✓^(28)^	✓^(29)^
Flood control	✓^(9)^			Urban forest				✓^(30)^	
Stormwater quality enhancement (nitrogen, phosphorus, coliform, total suspended solids)	✓^(10)^	✓^(10)^		Watershed, forest	✓^(31)^	✓^(32)^	✓^(33)^	✓^(34)^	✓^(35)^
Air purification/air quality regulation	✓^(11)^	✓^(11)^		Forests and other green spaces				✓^(36)^	✓^(37)^
Carbon sequestration	✓^(12)^	✓^(12)^	✓	Forests and other green spaces			✓^(38)^	✓^(39)^	✓^(40)^
Carbon storage	✓^(12)^	✓^(12)^	✓	Forests and other green spaces			✓^(41)^	✓^(42)^	✓^(43)^
Temperature regulation	✓^(13)^	✓^(13)^		Forests and other green spaces				✓^(44)^	✓^(45)^
Cultural									
Esthetic value	✓^(14)^			Forests and other green spaces				✓^(46)^	✓^(47)^
Recreation	✓^(15)^			Pocket parks, neighborhood parks, destination parks, regional parks	✓^(48)^	✓^(49)^	✓^(50)^	✓^(51)^	✓^(52)^
Educational opportunities	✓^(16)^	✓^(16)^		Forests, other green space, aquatic ecosystems, urban gardens, urban farms	✓^(53)^	✓^(54)^		✓^(55)^	✓^(56)^

*Production unit references* (1) Voicu and Been (2008), Farming Concrete (2010), Gittleman et al. (2010), and Ackerman (2012); (2) Peters et al. (2007), USDA (2007), and Peters et al. (2009); (3) Common knowledge: the notion that the majority of food arrives at NYC from great distances is already substantiated in 1913 (Miller et al. 1913); (4) USDA (2007); (5) New York Sea Grant (2001); (6) NYC Environmental Protection (2010a), New York City Department of Environmental Protection (2012), and Watershed Agricultural Council (2013); (7) New York State Department of Environmental Conservation (2010); (8) NYC Environmental Protection (2010a); (9) USDA Forest Service (2007) and NYC Environmental Protection (2010a); (10) NYC Environmental Protection (2010a); (11) U.S. Environmental Protection Agency (2001), Grove et al. (2006), Nowak et al. (2007), and McPhearson (2011), (12) Grove et al. (2006), Nowak et al. (2007), and McPhearson (2011); (13) Nowak et al. (2007), Rosenzweig et al. (2009), NYC Environmental Protection (2010b), and McPhearson (2011); (14) USDA Forest Service (2007) and Voicu and Been (2008); (15) New York City (2007) and New York City (2011); (16) Tidball and Krasny (2010) and McPhearson and Tidball (2013)

*Regulation, planning and management references* (17) NYC Soil and Water Conservation District (2013a); (18) Brannen (2011) and NYC Parks and Recreation; (19) East New York Farms! (2010), Farming Concrete (2011), Cohen et al. (2012), Harlem Grow (2012), The Battery Conservancy (2012), Added Value (2013), EcoStation: NY Inc. (2013), Food Systems Network NYC (2013), Green Guerillas (2013), and New York Restoration Project 2013a); (20) EPA Region 2 (2011) and Vintinner; (21) New York City Watershed Section Bureau of Water Supply Protection New York State Department of Health (2011); (22) Watershed Agricultural Council (2011); (23) New York City (2007) and NYC Environmental Protection (2010a); (24) New York State Department of Environmental Conservation (2010); (25) EPA Region 2 (2011); Vintinner; (26) New York City Watershed Section Bureau of Water Supply Protection New York State Department of Health (2011); (27) Watershed Agricultural Council (2011); (28) New York City (2007); (29) Riverkeeper (2013); (30) Rosenzweig et al. (2009) and New York City (2010); (31) New York City (2010); (32) New York City Department of City Planning (2002); (33) New York/New Jersey Harbor & Estuary Program (2011) and Catskill Watershed Corporation (2013); (34) New York City (2007, 2010), Mayor’s Office of Long-Term Planning and Sustainability, and New York City (2012) and NYC Soil and Water Conservation District; (35) NY/NJ Baykeeper (2009), Stormwater Infrastructure Matters (2010), Brown and Lipscomb (2011), Bronx Council for Environmental Quality (2013), Bronx River Alliance, and Newtown Creek Alliance; (36) MillionTreesNYC (2012); (37) New York Restoration Project (2013a) and Sustainable South Bronx (2013); (38) Regional Greenhouse Gas Initiative (2013); (39) MillionTreesNYC (2012); (40) New York Restoration Project (2013a); (41) Regional Greenhouse Gas Initiative (2013); (42) MillionTreesNYC (2012); (43) New York Restoration Project (2013c); (44) MillionTreesNYC (2012); (45) New York Restoration Project (2013c); (46) Sustainable South Bronx (2013); (47) NYC Parks and Recreation; (48) Voicu and Been (2008) and New York Restoration Project (2013a); (49) United States National Park Service (2003); (50) State of New York Department of Environmental Conservation (2009); (51) Flores et al. (1998); (52) New York City (2007), City of New York Parks and Recreation (2012); (53) Central Park Conservancy (2011), Friends of the High Line (2012), Bronx Council for Environmental Quality (2013), Eastern Queens Alliance (2013), Friends of Brook Park (2013), Sustainable South Bronx (2013) and Bronx River Alliance; (54) New York Restoration Project (2013b); (55) NY Department of Environmental Conservation (2013); (56) NYC Department of Education (2013) and NYC Parks; (57) Lower East Side Ecology Center (2009), Added Value (2013), Eastern Queens Alliance (2013), EcoStation: NY Inc. (2013), Friends of Brook Park (2013), Hudson River Foundation (2013), New York Restoration Project (2013b), Bronx River Alliance and North Shore Waterfront Conservancy of Staten Island

### Stormwater Quality Enhancement and Flood Control

Stormwater quality enhancement and flood control in NYC are provided at the local scale. Flood management is primarily within the purview of the city government, whereas stormwater quality is managed at the local, regional, and federal levels. Managing stormwater quality and quantity during heavy rain events has been particularly challenging for NYC given the legacy of its combined sewer overflow (CSO) system which, due to its limited capacity, discharges tens of thousand millions of gallons of contaminated water into local rivers and streams each year (Plumb 2006), causing significant eutrophication (Howarth et al. 2000), and limiting recreation. Almost two-thirds of NYC’s sewer system is built as a combined system that collects both stormwater runoff and municipal wastewater. During heavy precipitation events, the storm sewers overflow into the sanitary sewers, mixing stormwater and untreated sewage (as combined sewage overflows, or CSOs), and releasing them into local waterways. Despite continuing efforts to manage runoff, CSO overflows contaminated with coliform bacteria, organic matter, heavy metals, and other hazardous materials are discharged every year into the city’s receiving waters, a major reason why NYC’s tributaries do not meet Clean Water Act water quality standards for fishing and recreational use. The federal Environmental Protection Agency (EPA)’s Region 2 is responsible for administering the US Clean Water Act of 1972, which sets water quality standards for bodies of water within New York State including the Hudson River and New York/New Jersey Harbor. EPA has encouraged cities to use green infrastructure as a component of CSO programs, and adopted guidelines for implementation (NYC Environmental Protection 2010b). Although federal, state, and regional agencies have been more directly concerned with managing NYC’s stormwater quality than flooding, efforts to improve stormwater quality, particularly those which utilize green infrastructure, often involve reducing the volume of stormwater runoff and, therefore, have a positive spillover effect of reduced flooding.

**Fig. 2:**
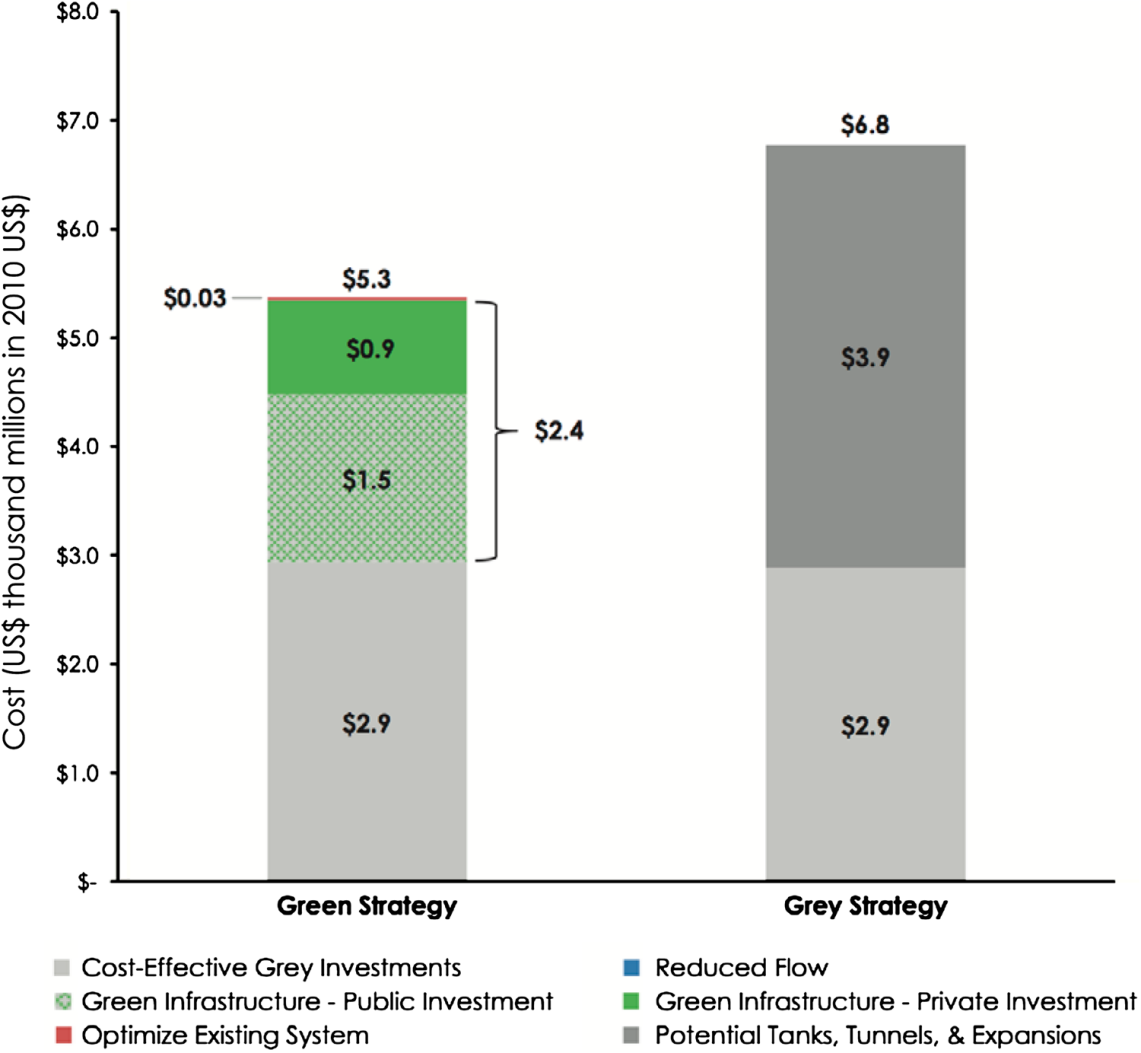
NYC Green Infrastructure Plan. Cost effectiveness of the private–public green strategy adopted by the city as compared to a gray infrastructure strategy. Image used with permission (NYC Environmental Protection 2010b)

In its 2007 PlaNYC, the City adopted a green infrastructure approach that attempts to simultaneously address the management of CSOs, as well as meet other goals, including improving urban green and open spaces and reducing greenhouse gas emissions (New York City 2007). NYC has dedicated US$2.4 thousand million (US$1.5 thousand million through public investment and US$900 million through private investment) to increasing and improving urban green infrastructure for stormwater absorption (NYC Environmental Protection 2010b; Cohen and Ackerman 2011). Green infrastructure investment is managed by NYC’s Department of Environmental Protection (DEP), which has created a number of innovative green infrastructure programs to transform impervious roofs, vacant lots, and streets into spaces that will absorb stormwater and prevent water pollution, while also providing habitat for biodiversity. DEP will invest US$187 million over the next 4 years for the installation of “blue roofs” that hold rainwater, large-street tree planters, “green streets,” porous concrete-paved parking lots, and gardens in paved vacant lots. The approach combines both small and large-scale green infrastructure development to control stormwater runoff. Two major measurable goals defined in the plan include (1) reduce CSO volume by 2 thousand million gallons per year and (2) install precipitation retention to manage storm events using green infrastructure on 10 % of impervious area across the CSO watershed by 2030 (New York City 2008, 2010). Using scenario analysis, the City estimated that by integrating green infrastructure into its stormwater management system, it could achieve a greater CSO volume reduction in a more cost effective manner than by relying solely on gray infrastructure (Fig. 2). The estimated aggregate annual value of new green infrastructure development including air quality improvement, CO_2_ reduction, energy savings, and increased property value is US$3145–US$5851 per hectare.

The Waterfront Revitalization Program (WRP) is the primary mechanism through which the City manages development of coastal and wetland areas. Originally adopted in 1982 and revised in 1999, it implements the City’s coastal planning obligations delegated by the State under the federal Coastal Zone Management Act. It includes policies to protect and restore tidal and freshwater wetlands in a way that maintains high filtration efficiency, manages direct and indirect discharges to water bodies, and minimizes property loss due to flooding through wetland and natural areas development (New York City Department of City Planning 2002). Because the program is based on the federal Coastal Zone Management Act, stakeholders at multiple levels of government—including the US Department of Commerce, NYC Department of State and Council of the City of New York are involved.

In addition to government agencies, community groups and non-profits such as the North Shore Waterfront Conservancy of Staten Island, Stormwater Infrastructure Matters (S.W.I.M.), Bronx Council on Environmental Quality, Sustainable South Bronx, Newtown Creek Alliance, New York-New Jersey Baykeeper, and Riverkeeper promote efforts that use green infrastructure to enhance water quality and protect waterfront communities from sea level rise, storm surges, and flooding. For instance, the Newtown Creek Alliance supports investment in green infrastructure, bioremediation, and habitat restoration to restore the ecological functions of the waterway. Additionally, the New York-New Jersey Harbor Estuary Action Plan for 2011–2015 is a regional-scale strategy to mitigate pathogens, toxics, nutrients, and floatable debris in the estuary, in part by supporting green technology that minimizes stormwater runoff (New York/New Jersey Harbor & Estuary Program 2011).

Due to the city’s CSO challenges, most of the effort to manage NYC’s stormwater has been undertaken by City agencies. This effort is a response not only to federal regulation, but also comes out of the recognition that the city’s surrounding waters can be a source of recreation and enjoyment for residents. These efforts are significantly supported by an array of actors at broader and more local scales.

### Drinking Water Supply and Quality

The supply and quality of drinking water are examples of provisioning services fully supplied at the regional scale and managed by multiple agencies and stakeholders at the federal, state, regional, and city scales. Over the last 20 years, NYC has engaged in an urban–rural partnership to protect the quality of its drinking water using ecological processes, thus avoiding costly water filtration infrastructure. Between 1830 and 1905, the City was able to secure access to pristine water from far northern areas of the Catskill–Delaware (Cat–Del) watershed rather than relying on local water sources which, at the time, would have been less costly (Appleton 2002). By the 1980s, NYC was receiving 90 % of its water from the Cat–Del and 10 % from the Croton watershed, east of the Hudson River. As farming became less financially viable, farmers in the Catskills began using increasingly intensive agricultural practices and concentrated livestock management. These practices resulted in elevated levels of polluted runoff and soil erosion. Meanwhile, as the value of agricultural land declined, the landscape began to transition from farms to residential development for vacationers and exurbanites, also leading to declining water quality. The combination of suburbanization in the Croton watershed and increasingly intensive agricultural practices in the Cat–Del watershed threatened NYC’s drinking water quality, compelling the City to engage in comprehensive watershed planning in the Cat–Del. Since 1991, the EPA has determined that the City is exempt from filtration requirements under the Surface Water Treatment Rule (part of the 1986 Safe Water Drinking Act Amendments). However, when the City applied for its second filtration waiver in 1993, a major component of its watershed plan involved land acquisition. This approach raised concerns among watershed residents about how the City’s watershed plan would affect the local economies and rural character of their communities. In 1997, the City, State, EPA, and local representatives from towns, counties, and environmental groups within the watershed signed the Watershed Memorandum of Agreement, which provides funding for economic and environmental programs including a regional economic development fund and a regional advisory group for water quality initiatives and watershed concerns (New York City Watershed Memorandum of Agreement 1997).

Although some elements of the City’s overall program have met with contention from upstate communities concerned with the economic impact of conservation programs, the City’s engagement with the farming community in protecting water quality has been largely perceived as positive (Pires 2004). A notable element of the City’s approach to comprehensive watershed planning is that it involved outreach initiatives resulting in farmer-developed solutions. A voluntary program called Whole Farm planning arose, in which the Department of Environmental Protection funds the Watershed Agricultural Council to provide technical staff to work with farmers in custom designing pollution control measures which are heavily informed by farmers’ own first-hand experience and knowledge (New York City Department of Environmental Protection 2012; Watershed Agricultural Council 2013). The program also provides participating farmers with a small stipend and exemption from water quality regulations. As of September 2007, 95 % of commercial farms in the Cat–Del were participating in Whole Farm (U.S. Environmental Protection Agency 2010) and the program was estimated to cost an eighth of what water filtration would have (Appleton 2002). Today the Cat–Del watershed provides 100 % of the drinking water used by the 8 million residents in NYC and one million residents of Westchester, Putnam, Ulster, and Orange counties.

Because of the watershed’s integrity and undisturbed natural water filtration system, NYC is one of five large cities in the country with a surface drinking water supply having such high quality that filtration is not required (NYC Environmental Protection 2010a). This payments-for-ecosystem-services approach suggests that investing in ecosystem services does not necessarily constitute a tradeoff between the needs of landowners and downstream resource users. Outreach processes that not only *inform*, but are also *informed by* program participants can lead to implementation programs that meet multiple stakeholder objectives. This innovative program has been successful in providing high-quality drinking water to NYC residents and is an example of how coordination among regional stakeholders can save money and protect critical ecosystem services for urban residents.

### Food Provisioning

While it is widely acknowledged that most of the food consumed by NYC residents is produced at the global scale, much of the city’s food is also produced at the regional and local scales. Little data are available on the direct relationship between production and consumption of food at the regional scale, but Peters et al. (2007, 2009) estimate that 34 % of produce and crops could potentially be supplied by agriculture production within the state. Seafood products are partially produced within the state. It is estimated that 13 % of seafood purchased by Fulton Market, the largest wholesale fish market in the region, are provided by New York State fisherman and other NY suppliers, while 67 % comes from other US states and 20 % from foreign sources (New York Sea Grant 2001).

NYC is facing critical challenges regarding food access, particularly with respect to availability and affordability of healthy food. Re-localization or regionalization of food production is argued to be an important part of the effort to make urban regions more sustainable and resilient by diversifying regional agriculture and providing urbanites access to fresh, healthy food (Kloppenburg et al. 2000; Clancy and Ruhf 2010). According to the NYC Coalition against Hunger, an average of 1.5 million New Yorkers, 25 % of whom are children, currently live in food-insecure households (NYC Coalition against Hunger 2013). Diseases linked to nutrition are on the rise in NYC, particularly among low-income individuals. More than half of NYC residents are overweight or obese and life expectancy in NYC’s poorest neighborhoods is eight years less than in its wealthiest (NYC Department of Health and Mental Hygiene 2004). In 2008, the Housing Economic and Infrastructure Planning division of the Department of City Planning conducted a survey which found that approximately three million New Yorkers who live in areas with low levels of fresh food purveyors have the highest diet-related diseases and the largest populations with low access to fresh foods based on income levels and other factors. The least healthy food environments have been found in East and Central Harlem and North and Central Brooklyn, areas with the highest proportions of Black residents and the lowest median household incomes (Gordon et al. 2011).

Although there has been relatively little public sector effort to supply New Yorkers with food produced at the regional scale, the Resource Conservation and Development for Wholesale Markets program is a partnership among the USDA, NYS Department of Agriculture & Markets, and the Lower Hudson/Long Island Resource Conservation & Development Council to develop water and rail infrastructure to transport agricultural products from the region into the city (NYC Soil and Water Conservation District 2013a). Food is also brought into the city directly from regional farms in a variety of ways including through Greenmarket, a non-profit network of 54 farmers markets offering food products from over 200 regional farms and fisherman, as well as many Community Supported Agriculture operations. By establishing CSA membership programs, non-profit organizations such as the Food Bank for New York City and Just Food connect regional food production to underserved communities in a way that is accessible and affordable.

Though only a small fraction of locally consumed food is produced in the city (Gittleman et al. 2010; Cohen and Ackerman 2011), the growing local urban agriculture movement is a promising trend in the development of urban ecosystem services. Food is produced in urban gardens in private homes, community gardens, rooftop gardens, and urban farms. In addition, these sites provide other ecosystem services such as runoff retention, habitat to support biodiversity, recreation and education opportunities, support sense of place, and are sites for social–ecological memory (McPhearson and Tidball 2013). The diverse NYC local food movement is comprised of City agencies, community groups, NGOs, research and education institutions, and many individuals. Although the City has not initiated comprehensive planning for the NYC food system, Five Borough Farm, a project spearheaded by the Design Trust for Public Space and in partnership with NYC Department of Parks and Recreation provides a city-wide roadmap for increasing NYC’s food production capacity through the City’s Green Infrastructure Program, the Parks Department’s GreenThumb program, rooftop agriculture, and other initiatives (Cohen et al. 2012). Connecting communities to land and other resources is one approach to increase a supply of healthy and affordable food. The City Council has passed a number of laws and resolutions to facilitate urban farming, including waiving height restrictions for rooftop greenhouses and creating an online database of city-owned property that indicates the land suitability for urban agriculture (Brannen 2011). GreenThumb, a Parks Department program, as well as two non-profits—Green Guerillas and NYC Restoration Project—provide resources for community gardens across the city (Green Guerillas 2013; New York Restoration Project 2013a). In addition, the City’s school gardens program—Grow to Learn—was developed in part to help combat unhealthy eating habits by familiarizing children with healthy fruits and vegetables (NYC Department of Education 2013). Meanwhile, a myriad of local community groups and other non-profits operate urban farms and use agriculture as a way to provide educational, economic, and broader community benefits (EcoStation: NY Inc.; Farming Concrete 2011; The Battery Conservancy 2012; Added Value 2013).

### Recreation

Green infrastructure in the city and region provides a number of cultural services to NYC. At the federal level, the National Parks Service manages parts of Jamaica Bay Wildlife Refuge for biking, birding, fishing, gardening, and other recreational activities (National Park Service 2013). NYC’s park system offers numerous recreational opportunities to residents in large-urban parks such as Central Park in Manhattan and Prospect Park in Brooklyn, as well as in playgrounds, sport fields and small pocket, and neighborhood parks. While the city’s park system is one of the largest in the world, PlaNYC (New York City 2007) acknowledges that many communities still lack sufficient access to parkland and open space. Therefore, the City has set a target of 0.6 ha of open space per 1000 residents, coupled with the goal of having a park located within a 10-min walk for all city residents. To achieve these goals, the City has committed to expand the park system by 1093 ha, improving existing facilities and offering extended hours in various park facilities with US$400 million slated for investment in the creation of new regional parks within the city boundaries (New York City 2007, 2011). Since 2007, more than 250 000 New Yorkers have gained 10-min walk access to a park, nearly 180 Schoolyards to Playgrounds sites and 260 green streets have been developed (New York City 2011). Schoolyards to Playgrounds is a partnership program among the Parks Department, Department of Education, and the non-profit Trust for Public Land, which makes schoolyards available to the public (City of New York Parks and Recreation 2013). Additionally, since the first Waterfront Plan in 1992, NYC has acquired 506 ha of waterfront as parkland. Wastewater treatment initiatives, including a US$6 thousand million allocation to upgrade the City’s wastewater treatment plants and more than US$1 thousand million to reduce CSOs, have contributed toward making the city’s waterways cleaner than they have been in a century and enhancing their recreational utility. The 2010 Waterfront Open Space Plan calls for dozens of redevelopment sites to be completed by 2020 (NYC Comprehensive Waterfront Plan 2011). In addition to City agencies and the Trust for Public Land which focus on improving recreational services throughout the city, a wide variety of community groups work to enhance recreational services at the community level, and as such, these groups’ missions tend to include multiple community-related goals such as environmental justice, safety, economic development, and improved air quality. Sustainable South Bronx, for example, has played a significant role in the development of the South Bronx Greenway, and advocates for environmental justice in a community that is disproportionately impacted by poor air quality due to vehicle traffic and power plant emissions (Sustainable South Bronx 2013). Other community groups working to improve recreation and other services at the neighborhood or site level include Friends of Brook Park (also in the South Bronx), Rockaway Waterfront Alliance, Bronx Council on Environmental, Friends of the High Line, and Eastern Queens Alliance (Friends of Brook Park; Friends of the High Line 2012; Bronx Council for Environmental Quality 2013; Eastern Queens Alliance Inc.; Rockaway Waterfront Alliance). Although there are fewer initiatives aimed at enhancing New Yorkers’ access to recreational services at the regional level, the Regional Plan Association’s Greensward Campaign envisions linking urban green spaces with large-scale regional natural reserves to form a coherent green space system and address relationships among economy, social equity, and the environment (Flores et al. 1998). At the state level, the New York State Office of Parks Recreation and Historic Preservation manages 178 state parks and 35 historic sites (State of New York Department of Environmental Conservation 2009), though the published literature on the extent to which NYC residents use these recreational lands is not available. Overall, however, most management strategies around recreational services specifically for city residents described in the literature focus on providing opportunities in close proximity to where people live, either through strategic city-wide planning or neighborhood and site-level projects.

Despite these efforts, over 1.5 million New Yorkers live more than a 10-min walk from a park and underserved areas are disproportionately located in Queens, Brooklyn, and Staten Island. The Mayor’s Office of Environmental Coordination has also identified underserved city neighborhoods of high population density that are far from parkland and have low park density. Twenty-four of these underserved areas are in Brooklyn neighborhoods, 21 in Queens, yet only four in Manhattan and three in Staten Island (NYC Mayor’s Office of Environmental Coordination 2013). The milieu of community-based organizations in NYC with social equity and environmental missions whose programs address recreation are a reflection of this disproportionate access. Thus, overall provisioning as well as equitable provisioning of recreational services remains key challenges for the city.

### Relationship Between the Scale of Ecosystem Service Production and Management

Federal mechanisms regulate and influence how the City manages some of its most important ecosystem services, including water supply and stormwater quality. Through its Americorps program, the federal government is also involved in enhancing educational opportunities facilitated by ecological processes and functions (New York Restoration Project 2013a). Regional partnerships play a more (in the case of drinking water quality) or less (in the case of recreation services) pivotal role in ecosystem services provisioning to city residents. Moreover, to some extent, city agencies are involved in managing and designing policy and planning for almost all of the ecosystem services that we reviewed and community groups play a significant role in neighborhood and site-level project management. Local community groups and other non-profits tend to play a role in almost every ecosystem service consumed in NYC, with the exception of flood control and water supply.

Depending on the scale of production, our review finds variation in the dominant management scale (Table 1). Drinking water supply and quality enhancement, produced at the regional scale, are largely managed through regional-scale cooperation and partnerships at all levels. Stormwater quality enhancement and flood control, produced at the local scale, primarily involve city-level efforts in partnership with agencies at the regional, state, and federal levels. Recreation, also produced at the local scale, is mostly planned for and managed at the city and community scales. These services exhibit a match between the scale of production and scale of management, and in the cases of drinking water supply, drinking water quality enhancement and stormwater quality enhancement, management at multiple scales.

On the other hand, a number of services exhibit a mismatch between the scale at which they are produced and the scale at which they are managed, regulated, or planned for. Efforts to ensure that New Yorkers have access to a supply of healthy and affordable food largely happen through community groups and non-profits in NYC, and to a lesser extent, city agencies and regional actors. However, the vast majority of food consumed by New Yorkers is not produced within the region and city. Many of the planning and management efforts around food access and affordability are aimed at localizing production, which would lead to a better alignment between production and management scales. Similarly, although air purification, carbon sequestration, carbon storage, temperature regulation, and food are all produced at the local, regional, and global scales, with the exception of the CO_2_ offset allowance for afforestation projects which is part of the Regional Greenhouse Gas Initiative (Regional Greenhouse Gas Initiative 2013), these services appear to be largely managed at the municipal level through efforts such as MillionTreesNYC, aimed at expanding NYC’s urban forest (McPhearson 2011), and local-scale greening and urban agriculture initiatives. More regional, state, and federal level efforts as well as better coordination between broad and local-level agencies are needed to produce and enhance C sequestration, C storage, temperature regulation, and food production.

## Discussion

### Why Scale Issues are Important

We find that ecosystem services consumed by New Yorkers are produced at the local, regional, and global levels, and managed by local non-governmental actors as well as governmental actors at all levels. These actors have been highly successful at maintaining a supply of clean water for the city, but generally less successful at managing flooding and stormwater runoff, maintaining a supply of healthy and affordable food for all residents, and ensuring equitable access to recreation in green spaces throughout the city. Notably, recreation and food can, in some ways, be more accessible when provided at the local scale if, for example, gardening opportunities are available for low-income households. Moreover, stormwater management is inherently produced at the local scale, since the conditions which create flooding and overflow mostly occur within relatively localized watersheds. That producing these services locally has been so challenging may be related to densification, which decreases land availability and puts increased demands on the use of what land is available. By contrast, management of the city’s water supply is unrelated to land use within the city. Although competing land use interests were at issue within the water supply watershed region, a greater availability of land resources may have placed fewer political pressures on compromises that were made in order to develop Whole Farm, the City’s land acquisition program, and local land use plans that limit real estate development.

Efforts to protect the quality of NYC’s drinking water may have also been successful due to the coordination that has occurred among so many actors at different organizational and jurisdictional levels. In addition to farmers’ participation in Whole Farm planning, in order to reduce the cost of water purification, the City needed to engage town and county planners in efforts to restrict real estate development that can also be a significant contributor of non-point source pollution. These efforts aligned with not only City interests, but also the interests of state agencies responsible for administering the federal Safe Drinking Water Act and those of regional environmental groups. Coordination among regional and state-level agencies (which have jurisdiction over the scale at which drinking water is produced) and city-level agencies (which have jurisdiction over the scale at which drinking water is consumed) has likely been a major reason why the quality of NYC’s drinking water remains so high. A myriad of organizations are working toward building a local food system in the city, and Five Borough Farm has provided a starting point for comprehensive urban agriculture planning. However, the City has yet to design an over-arching strategy for supporting, harnessing the resources of, and coordinating these actors.

### Utility of the Ecosystem Services Framework

Though the ecosystem services framework has utility for uniting biodiversity conservation goals with goals for human health and well-being and illuminating ways in which ecosystem functions benefit people in urban settlements, it is difficult to operationalize. The framework does not help to resolve the problematic ways in which spatial mismatches exist between the locations where ecosystem services are supplied and where they are consumed or demanded. Inherent difficulty coordinating management and planning across government jurisdictions and neighborhood institutions is exacerbated both by potentially competing goals among these groups, and by scale mismatches in supply and demand. These concerns suggest the need for further research to better understand the obstacles and potential solutions for employing the ecosystem services framework to achieve urban resilience and sustainability goals.

## Conclusion

Meeting the sustainability and resilience goals within existing policy and plans in NYC will require a better understanding of the current and predicted future state of biodiversity and ecosystem services in a megacity undergoing change. Here we review key urban ecosystem services of high priority in the city in order to provide a baseline for future comparison and to generate discussion about how policy, planning, and management may be improved to transition NYC to a more sustainable and resilient city. Future environmental change, including land use transformation driven by population expansion and development, continued risk from invasive non-native species, and regional climate change place mounting pressure on existing biodiversity in the city, and, therefore, pose a threat to the ecosystem services upon which urban residents rely. Providing services which are inherently produced at the local scale, or lead to more equitable access when produced locally, has been a significant challenge in NYC, particularly with respect to recreation, food provisioning, and stormwater management. We suggest that better coordination among stakeholders and adaptation of land use planning to meet urban residents’ needs in the context of increasing densification are needed to support health and well-being.

We find a number of important research questions remain. First, a comprehensive citywide assessment of the current state of ecosystem services production by urban green and blue infrastructure in the city would enable planners and managers to consider how ecosystem services can be improved and where tradeoffs exist. Though existing policies and plans in NYC have multiple ecosystem services goals, it is unclear how a particular management strategy will result in greater synergy and decreased tradeoffs among potentially competing ecosystem service goals. Additional research is needed to understand the inequalities driven by mismatches between the spatial distribution of the supply of ecosystem services and the spatial distribution of the demand for ecosystem services, especially in underserved areas of the city. For example, a recent effort to map the social need for ecosystem services around vacant lots in NYC found that low income, high population density areas of the city also tend to have decreased access to green space where many ecosystem services are produced (Kremer et al. 2013; McPhearson et al. 2013).

More direct inclusion of biodiversity conservation principles into governance practices and sustainability and resiliency policy initiatives could provide opportunities for collaboration between the biodiversity research community and natural resource managers and planners. Still, further study is needed, since the processes underlying patterns of biodiversity in cities and how they influence ecosystem services production are poorly understood (Faeth et al. 2011). Understanding the human-controlled and natural processes that alter urban biodiversity and ecosystems is essential for managing and planning for future delivery of ecosystem services. However, we still know little about the relationship between biodiversity, urban ecosystem processes, and ecosystem services in cities. Despite the need for additional research, we find that ecosystem services framework provides a utilitarian approach to motivate urban biodiversity conservation, promoting human–nature interactions in cities, and highlighting the value of ecosystems to promoting livable, resilient cities.
